# High level *MYC* amplification in B-cell lymphomas: is it a marker of aggressive disease?

**DOI:** 10.1038/s41408-019-0271-z

**Published:** 2020-01-13

**Authors:** Priyanka A. Pophali, Lisa M. Marinelli, Rhett P. Ketterling, Reid G. Meyer, Ellen D. McPhail, Paul J. Kurtin, Raphael Mwangi, Matthew J. Maurer, Thomas Habermann, Rebecca L. King

**Affiliations:** 1 Division of Hematology, Department of Medicine, Mayo Clinic, Rochester, MN, USA; 2 Division of Hematopathology, Department of Laboratory Medicine and Pathology, Mayo Clinic, Rochester, MN, USA; 3 Division of Laboratory Genetics and Genomics, Department of Laboratory Medicine and Pathology, Mayo Clinic, Rochester, MN, USA; 4 Department of Health Sciences Research, Mayo Clinic, Rochester, MN USA

**Keywords:** Cancer genetics, B-cell lymphoma

## Abstract

While *MYC* translocations in B-cell lymphoma (BCL) have been extensively studied, the significance of *MYC* amplification (*MYC* amp) is poorly understood. This study characterizes BCL showing *MYC* amp, defined as uncountable FISH signals. Retrospective analysis of all BCL FISH for *MYC* aberrations performed at our institution (1/2010–2/2018) identified 44/9715 (0.45%) cases with *MYC* amp. *MYC* amp probe signals appeared in a cloud-like distribution (70%) or in a single homogenous-staining-region (30%). In total 59% also had *MYC* separation by breakapart probe indicating concurrent *MYC* translocation. The most common morphology was large cell (82%) and diagnosis was diffuse large BCL (DLBCL, 50%). In total 88% were germinal center B-cell-like by Hans algorithm. In total 12/42 (29%) cases were “double-hit” by WHO criteria (DHL/THL) in addition to having *MYC* amp. The estimated 2-year overall survival (OS) of DLBCL cases with *MYC* amp was 80%. There was no significant difference in OS between DLBCL and DHL/THL among cases with *MYC* amp, suggesting a poor prognostic impact of MYC amp. However, when compared to a larger cohort of DLBCL and DHL/THL, *MYC* amp did not have prognostic significance. In summary, *MYC* amp in BCL is rare, most commonly occurs in DLBCL, and was not associated with survival in our cohort.

## Introduction

*MYC* is a multifunctional transcription factor that plays a significant role in tumorigenesis^1^. *MYC* rearrangements are characteristic of several types of B cell neoplasms. Diffuse large B cell (DLBCL) and high grade B cell lymphomas (HGBL) demonstrating *MYC* translocation with concurrent *BCL2* and/or *BCL6* rearrangements, colloquially called double and triple hit lymphomas (DHL and THL), have poor clinical outcomes^2^. The prognostic implications of DHL and THL underlie the 2017 World Health Organization (WHO) update, which includes the cytogenetically defined category of “High grade B cell lymphoma with *MYC* and *BCL2* and/or *BCL6* rearrangements” (DHL/THL) as its own distinct entity^3^.

According to the WHO, for prognostic and classification purposes, *MYC* copy number increase or amplification (amp) is not considered equivalent to MYC rearrangement and remains of uncertain significance. Cases without *MYC* rearrangements should be diagnosed as diffuse large B cell lymphoma (DLBCL) or high grade B cell lymphoma, not otherwise specified (HGBL, NOS)^3^. However, in BCL that do not meet the WHO criteria for DHL/THL, FISH often detects increased *MYC* copy numbers, either due to additional copies of chromosome 8 (thus including *MYC*) and/or structural abnormalities of chromosome 8 resulting in *MYC* gene duplication or gene amplification. These types of copy number increases are fairly common in DLBCL and are seen in ~30–40% of cases^4^. The significance of these low-level copy number gains (3–10 copies per cell) is controversial. While some studies have correlated increased *MYC* copy numbers with increased protein expression and disease aggressiveness^4,5^, other studies report no significant prognostic impact^2,6^, albeit with small sample sizes and increased copy number typically defined as anything greater than 2 copies per cell^2,5–10^.

*MYC* amplification (*MYC* amp), however, is well established to play a diagnostic and prognostic role in several other malignancies, including medulloblastoma, chondrosarcoma, and post-radiation cutaneous angiosarcoma, in which it imparts a poor prognosis^11–14^. Within our FISH practice, we rarely observe cases of BCL exhibiting high level *MYC* amp (uncountable signals) that from a cytogenetics perspective is distinct from the low-level copy number changes seen in most BCL. “Uncountable” is described by the International System for Human Cytogenetic Nomenclature (ISCN) as being representative of cases in which the “number of signals cannot be quantified because it is increased in a copy number beyond that which can be reliably counted”^15^. Due to its rarity, as well as variable definitions used for calling *MYC* amp by FISH in BCL, both reporting and studying these cases is challenging. Since *MYC* amp is a significant negative prognostic factor in other malignancies, and there is a suggestion that low-level *MYC* copy number changes may play a similar role in BCLs, we hypothesized that high-level *MYC* amp (defined as above) would have a negative effect on outcomes in DLBCL. The primary objective of this study was to review the cytogenetically distinct set of cases in which *MYC* FISH revealed *MYC* amp, and to characterize the pathologic, cytogenetic, and clinical features of this rare finding.

## Methods

**Fig. 1:**
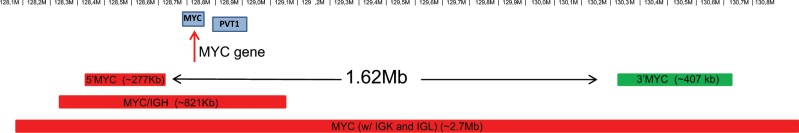
Genomic location of *MYC* gene versus the various *MYC* FISH probes used for evaluation in B-cell malignancies. Genomic View of the *MYC* gene region on chromosome 8q24.1 (*MYC* gene highlighted by Black Arrow). Located underneath are boxes representing the 3 sets of *MYC* probes used in this study: First Row: Break-apart 5′ (Red)/3′ (Green) probe; Second Row *MYC* probe (Red) from the *MYC*/IGH D-FISH probe set; Third Row: MYC probe(Red) from the *MYC*/IGL D-FISH and *MYC*/IGK D-FISH probe sets. The Red and Green boxes are in the appropriate genomic location and indicate the genomic footprint of the individual probe. The *MYC* BAP probe and *MYC*/IGH D-FISH probes are Abbott Molecular and represented here with permission. The *MYC*/IGL and *MYC*/IGK D-FISH probes are Mayo laboratory developed probes.

After receiving approval from the Institutional Review Board, and ensuring appropriate patient consent, we conducted a retrospective analysis of all BCL cases that had FISH testing within the Mayo Clinic cytogenetic database from January 2010 to February 2018. All FISH studies reported as *MYC* amp were re-reviewed by a cytogeneticist to verify the level of amplification and the *MYC* probe(s) involved in the amplicon. *MYC* FISH testing included *MYC* break-apart (BAP) and *MYC*/IGH dual-fusion (D-FISH) (both Abbott Molecular, Abbott Park, IL, USA); *MYC*/IGL D-FISH and *MYC/*IGK D-FISH (Mayo laboratory developed assay); *BCL2* BAP and *BCL6* BAP (Abbott Molecular) (See Fig. 1 for probe maps) using previously published laboratory methods and specimen-specific laboratory protocols^16^. Available hematoxylin and eosin (H&E) stained slides were reviewed by two independent hematopathologists to classify cases as either large cell or high grade. Definitions of high-grade and large-cell cytologic features are in accordance with WHO recommendations and detailed in a previous publication from our group^16^. Pathology reports were reviewed for CD10, BCL6, MUM1, BCL2, and MYC immunohistochemistry (IHC). When stains were not reported or not available for review, they were performed on unstained slides when available^16^. Cell of origin (COO) was determined using the Hans algorithm^17^.

There are no well-established criteria defining *MYC* amp in lymphoma. While six copies per cell have been used to define *MYC* amp in breast cancer, aneusomy is more common in lymphoma and frequently causes low-level copy number gains^18^. Here we distinguished high-level copy number gains from more common lower level gains which commonly stem from tumor tetraploidy^9^. This study sought to characterize the rare cases seen in our FISH practice with uncountable amplification of the *MYC* gene beyond what would be expected with chromosomal gain. “Uncountable” is described by the International System for Human Cytogenetic Nomenclature (ISCN) as being representative of cases in which the “number of signals cannot be quantified because it is increased in a copy number beyond that which can be reliably counted”^15^. In this study, *MYC* amplification was defined as uncountable amplification of the *MYC* gene.

Clinical information was collected through chart review. Cases within the Mayo Clinic system had electronic health records available. For cases that were sent to our referral laboratory from other centers, we contacted the referring provider with a request to obtain patient consent. Paper records for the patients who consented were then reviewed. In order to understand the prognostic impact of *MYC* amplification on overall survival in lymphoma, we compared the outcome of cases identified with *MYC* amplification from the cytogenetic database with diffuse large B-cell lymphoma (DLBCL) cases enrolled in the Mayo Clinic and University of Iowa Lymphoma SPORE Molecular Epidemiology Resource (MER)^19^. Survival analysis was performed using the Kaplan–Meier method and the Wilcoxon rank-sum test. All statistical analysis was performed using JMP 14.1.0 and R version 3.4.2.

## Results

### Pathologic features

FISH analysis for *MYC* aberrations identified 44/9715 (0.45%) cases with *MYC* amplification. Table 1 summarizes the pathologic and cytogenetic features of these cases. Irrespective of FISH results, the most common morphologic pattern was large BCL (LBCL) (82%; 33/40), followed by high grade BCL (13%; 5/40). Two cases were immunophenotypically and morphology compatible with plasmablastic lymphoma and were classified as such.

**Table 1:** Summary of pathologic and cytogenetic features of cases with MYC amplification.

Final Classification *n* = 42^a^	WHO DHL (*n* = 12)	DLBCL (*n* = 22)	HGBCL, NOS (*n* = 3)	Plasmablastic (*n* = 2)	Unknown Cytology^b^ (*n* = 3)
High-grade morphology	1/11 (9%)	0/22 (0%)	3/3 (100%)	NA	Unknown
GCB by Hans	8/9 (89%)	15/18 (83%)	2/2 (100%)	NA	Unknown
Double expresser	5/7 (71%)	6/16 (38%)	2/2 (100%)	1/1 (100%)	1/1 (100%)
MYC-non-IGH	10/12 (83%)	7/22 (32%)	1/3 (33%)	2/2 (100%)	1/3 (33%)
MYC-IGH	2/12 (17%)	2/22 (9%)	0/3 (0%)	0/2 (0%)	0/3 (0%)
MYC-any R; NO BCL2 or BCL6-R	NA	9/22 (41%)	1/3 (33%)	2/2 (2%)	1/3 (33%)
MYC-any R; BCL2-R or BCL6-R present	12/12 (100%)	NA	NA	0/2 (0%)	NA
NO MYC-R; BCL2-R and/or BCL6-R present	NA	5/21 (24%)	0/3 (0%)	NA	2/2 (100%)

Cases are classified by final WHO pathologic classification based on morphologic, immunohistochemical, and cytogenetic features

*NA* Not applicable

*GCB* Germinal center B cell

*BCL2-R*: BCL2 rearranged

MYC-any R = MYC sep = MYC any rearrangement

^a^Two cases are not included in the table as complete BCL2 or BCL6 FISH was not available for review precluding final classification as either DLBCL or HGBL NOS

^b^Unknown Cytology: cases in which H&E was not available for morphologic review, thus cannot be classified as either DLBCL or HGBL NOS

Of the 33 cases with LBCL morphology, Hans algorithm IHC was available for 26 cases: 23 (88%) were germinal center B-cell-like (GCB), and 3 (12%) were non-GCB. MYC IHC was available for 22 LBCL cases: 15/22 (68%) met the MYC positivity threshold of 40% or more; 6/22 (27%) cases had <40% *MYC* expression by IHC and only one case (5%) was negative for MYC staining. IHC for BCL2 was positive (≥50%) in 21/30 (70%) cases. Of the 21 cases with both MYC and BCL2 IHC, 9 (43%) were double expressers (DEL). The DELs included 5 GCB, 2 non-GCB, and 2 without available Hans IHC.

Among the HGBL, 2 cases each could be categorized as GCB and non-GCB by applying the Hans algorithm. In total 5/5 (100%) were MYC positive (≥40%) and 4/4 (100%) were BCL2 positive (≥50%). For the 2 plasmablastic BCL cases, MYC was positive and BCL2 was negative by IHC for one case; MYC unavailable and BCL2 positive for the other case.

### Cytogenetic features

**Fig. 2:**
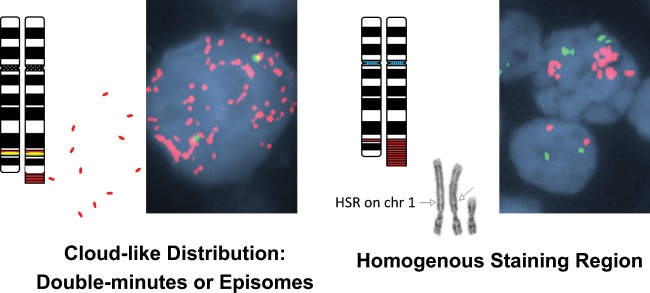
Representative images illustrating the two main patterns of *MYC* amplification. LEFT: Single Interphase nucleus demonstrating *MYC* amplification as a “Cloud-like” distribution or innumerable dispersed 5′ *MYC* signals throughout the interphase nucleus using the 5′ (Red)/3′(Green) break-apart *MYC* probe. The Chromosome 8 Ideograms demonstrate the location of the intact break-apart *MYC* probe at 8q24.1. The *MYC* amplification corresponds to the individual small fragments of red genomic material representing either Double-minutes or Episomal amplification of the 5′ *MYC* (Red) gene region. RIGHT: Single Interphase nucleus demonstrating *MYC* amplification as a Homogeneous Staining Region (HSR) or innumerable overlapping *MYC* gene signals confined to one or more specific locations in the interphase nucleus using the *MYC* (red) and IGH (green) D-FISH probe set. A single normal interphase nucleus is also present for comparison. The Chromosome 8 Ideograms illustrate the location of the *MYC* locus at 8q24.1 with a red probe and an example HSR on 8q. The G-Banded Chromosomes 1 from this particular case demonstrate one normal chromosome 1 and two copies of an abnormal chromosome 1 with an HSR on 1p (arrows) corresponding to the *MYC* amplification identified in the interphase nucleus.

*MYC* amp was seen in 95–100% cells in 39 out of 44 cases (89%). FISH probe signals exhibited two distinct patterns of distribution (Fig. 2). The most common pattern was a dispersed, cloud-like distribution (CLD) of possible double minutes or episomes in 31/44 (70%) of cases. Less commonly observed in 13/44 (30%) of cases, was a single, extra-large FISH signal or an “interphase homogenous staining region” (HSR), likely corresponding to innumerable gene copies aligned along the same chromosome. The probe sets tested and those in which the *MYC* probe was amplified in each case are illustrated in Table 2. Six cases had *MYC* amp on the D-FISH probe(s) but not in the BAP. Among 38 cases with amp in a *MYC* BAP probe, 21 (55%) had amp of the 5′ probe alone, 14 (37%) had amp of the intact BAP probe, 2 (5%) had amp of the 3′ probe alone and 1 (3%) case had an unusual pattern including amplification of both the intact BAP and distinct, separate amplification of the 5′ probe in the same cells.

**Table 2:**
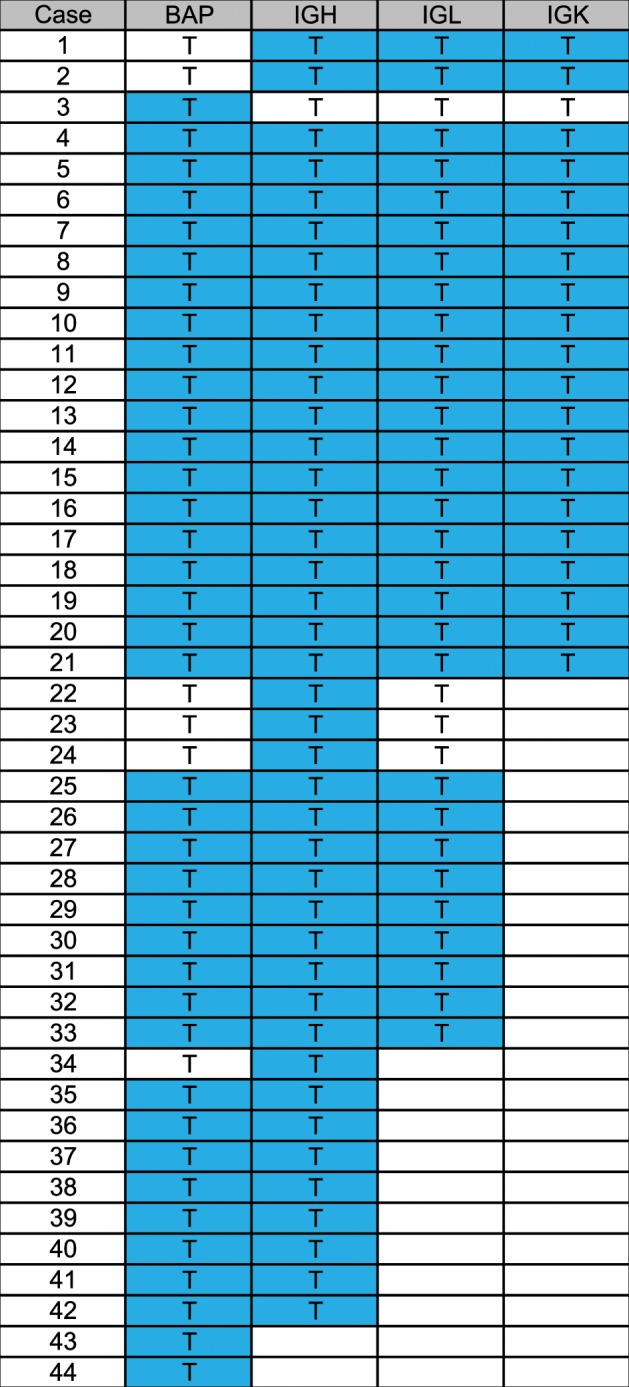
Summary of FISH probes tested in each case evaluated in the study.

Each row represents a single case and each column a *MYC* FISH probe set. T indicates that the probe was tested in that particular case, and blue shading indicates that *MYC* amplification was seen in that probe set. BAP: breakapart probe, *MYC*-IGH, -IGK, -IGL refer to dual-fusion probe sets for *MYC* and the respective partner gene

In total 26 of 44 (59%) cases with *MYC* amp also had *MYC* separation by BAP, suggesting a concurrent rearrangement of the *MYC* gene. Of these, 4 of 25 in which the IGH probe was also performed (16%) had *MYC*/IGH fusion, none had IGL (0/15) or IGK fusion (0/13). One case did not demonstrate *MYC* separation by BAP but had *MYC*/IGH fusion using the *MYC/IGH* D-FISH probe, a phenomenon previously described by our group^20^. In addition to *MYC* amp, *BCL2* rearrangements were seen in 16/43 (37%) of cases, and *BCL6* rearrangements were detected in 3/41 (7%). No cases had both *BCL2* and *BCL6* rearrangements. Overall, in addition to *MYC* amp, 12/42 (29%) cases had *MYC* rearrangement and BCL2 or BCL6 rearrangement, and thus met the WHO diagnostic criteria for DHL/THL irrespective of the presence MYC amp (Table 1). In total 7 of the 31 cases that did not meet the criteria for DHL/THL, had *MYC* amp with *BCL2* rearrangement.

### Final pathologic classification

The final pathologic classification of the cases with *MYC* amp accounting for morphologic, FISH and immunohistochemical characterization, as available, is presented in Table 1. In summary, of all 44 *MYC* amp cases, there were 12 DHL/THL using WHO criteria, 22 DLBCL, NOS, 3 HGBCL, NOS, and 2 plasmablastic lymphomas. An additional 5 cases could not be definitively classified because of lack of morphologic data (*n* = 3) or lack of complete FISH data to evaluate for a DHL/THL (*n* = 2). Within the largest cohort, DLBCL, NOS (*n* = 22), there were 5 cases in which *MYC* amp (but not rearrangement) was present with a *BCL2* rearrangement.

### Clinical features

Table 3 summarizes the clinical features of this cohort. The median age of all 44 patients was 66 (range 25–88) years, with 26 (59%) males and 18 (41%) females. Follow-up information was available for 36 (82%) of the 44 cases with *MYC* amp. In 26 (72%) of the 36 cases, *MYC* amp was identified at the time of initial diagnosis of aggressive lymphoma. Of these, 2 occurred in the post-transplant setting (post-transplant lymphoproliferative disorder), 1 was an HIV-associated plasmablastic lymphoma, and 4 cases were transformed from low grade lymphomas (2 each from chronic lymphocytic leukemia/small lymphocytic lymphoma (CLL/SLL) and follicular lymphoma (FL)). In 5 (14%) of the 36 cases, *MYC* amp was identified at the time of relapse with FISH results at initial diagnosis not known. Clinical details of presentation were not known in 5 other cases.

**Table 3:** Summary of clinical features cases with *MYC* amplification.

Clinical features^a^	
Age at diagnosis, median (range), years	66 (25–88)
Male: Female	1.4: 1
Bone marrow involvement	1/21 (5%)
Other extranodal site involvement	28/31 (90%)
Gastrointestinal	10/31 (32%)
Timepoint of sample studied with *MYC* amp	
Initial diagnosis	26/31 (84%)
Relapse	5/31 (16%)
LDH, median (range), U/L	314 (143–3942)
First line R-CHOP	17/26 (65%)
Duration of follow-up, median (95% CI), months	27.9 (18.4–45.6)
Deaths	16 (44%)
Cause of death-lymphoma relapse	10/16 (62%)

^a^*N* = 44 for age and M:F; *N* = 36 for other clinical parameters unless otherwise specified

Stage at presentation was limited (Ann Arbor stage I and II) in 12 cases, advanced (stage III and IV) in 13 cases and not known in 11 cases. Only 1/21 (5%) had bone marrow involvement which was noted to be focal. In total 28/31 (90%) cases had extranodal disease involvement with the most common extranodal site being gastrointestinal tract in 10 cases (32%). Lactate dehydrogenase was higher than the upper limit of normal for 13/18 (72%) cases with a median of 314 (range 143–3942) U/L. Due to missing information, International Prognostic Index could not be calculated for most of the cases, and therefore is not reported.

### Clinical outcomes

Treatment information was available for 26 of the 36 cases. The majority of cases received an anthracycline based regimen: 17/26 (65%) R-CHOP, 5/26 (19%) DA-R-EPOCH, 1/26 (4%) R-CODOX-M/IVAC and 1/26 (4%) R-Hyper-CVAD. One patient received rituximab-lenalidomide while another received single agent rituximab. Response to first line treatment was available in 25 cases: 16 complete remissions, 6 partial remissions, 2 progressive and 1 stable disease. Of the 16 patients who achieved CR, 4 (25%) patients had relapsed within 2 years. At a median follow-up of 27.9 (95% CI 18.4–45.6) months, 16 deaths had occurred: 10 lymphoma-related, 1 metastatic lung cancer, 1 infection, 4 causes not known. Of those who were alive at last follow-up, 3 patients were in relapse, 10 remained in first complete remission and status was unknown for 5.

**Fig. 3:**
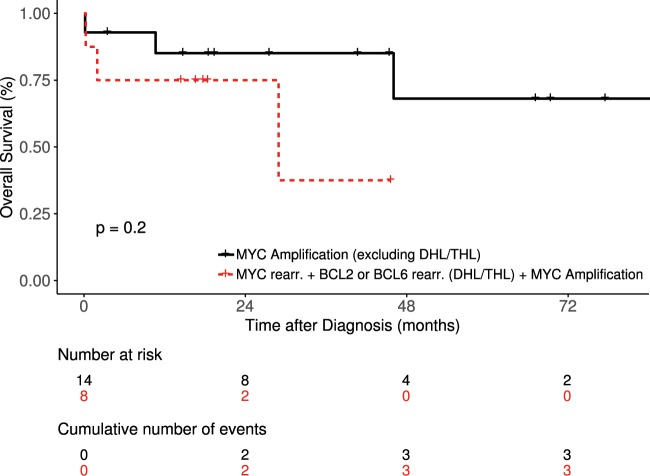
Overall survival of DLBCL patients (from initial diagnosis only) with *MYC* amp alone versus *MYC* amp with concurrent *MYC* rearrangement and *BCL2* or *BCL6* rearrangement.

To more accurately assess the impact of MYC amp on OS, we excluded 13 cases that would be expected to have poor outcomes: *MYC* amp at lymphoma relapse (*N* = 5), non-DLBCL morphology (*N* = 5; 3 HGBCL; 2 plasmablastic lymphomas) and transformation from CLL or FL with prior anthracycline use (*N* = 3) (Supplementary data). The 8 cases with DHL/THL in addition to *MYC* amp were analyzed separately and had an estimated 2-year OS of 75%. For the 14 cases with *MYC* amp on FISH at initial DLBCL diagnosis and not meeting WHO criteria for DHL/THL, the estimated 2-year OS was 80%. There was no significant difference in OS of *MYC* amp cases without (*n* = 14) vs with DHL (*n* = 8) (Fig. 3; median OS not reached vs 28.95 months, *p* = 0.2).

**Fig. 4:**
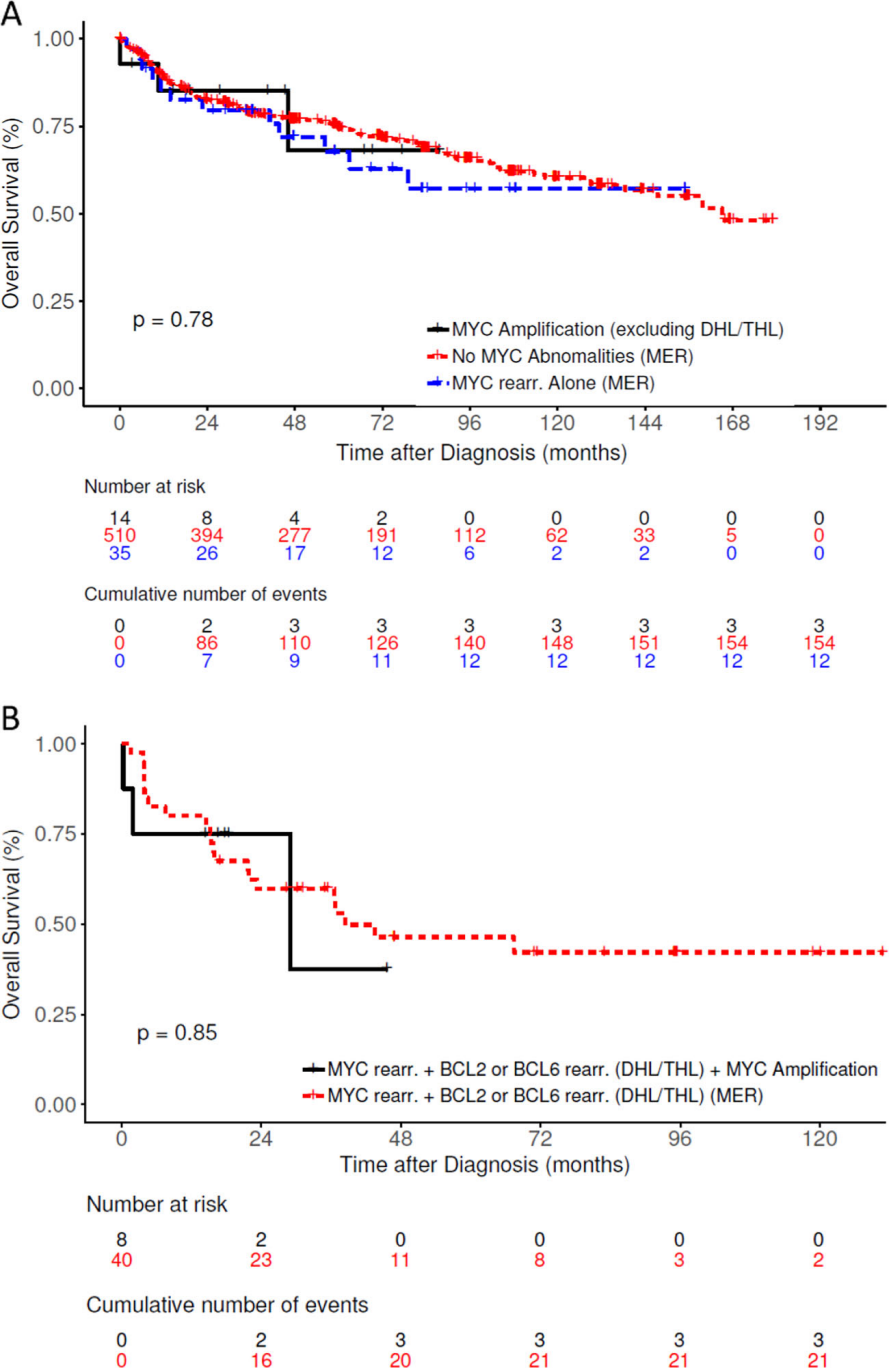
Overall survival of DLBCL patients from high-level *MYC* amplification cohort compared to DLBCL patients from the MER cohort. **a** High-level *MYC* amplification without DHL/THL versus no *MYC* abnormalities on FISH and *MYC* rearrangement alone (single hit) DLBCL cases from the MER. **b** High-level *MYC* amplification with DHL/THL versus DHL/THL cases from the MER.

Since we found no difference in OS between *MYC* amp cases with and without DHL, we hypothesized that the finding of *MYC* amp by FISH in DLBCL by itself may have a negative prognostic impact, similar to DHL. We proceeded to test this hypothesis by comparing the survival outcomes of our *MYC* amp cohort with cases from the MER categorized in the following groups by FISH findings– (1) DLBCL with no *MYC* abnormalities, (2) DLBCL with *MYC* rearrangement alone (single hit lymphoma, SHL) and (3) DLBCL with *MYC* rearrangements and *BCL2* or *BCL6* rearrangements (DHL/THL). There were no significant differences in OS of DLBCL cases with *MYC* amp compared to DLBCL with no *MYC* abnormalities and *MYC* SHL from the MER (*p* = 0.1; Fig. 4a). Similarly, there were no differences in OS of DHL with *MYC* amp versus DHL cases from the MER (*p* = 0.42; Fig. 4b).

There were no statistically significant differences in OS for the 14 *MYC* amp cases (not meeting criteria for DHL) by double protein expression (yes, *n* = 3 vs no, *n* = 5; *p* = 0.06), *MYC* amp pattern on FISH (HSR, *n* = 6 vs CLD, *n* = 10; *p* = 0.57), additional presence of *MYC* rearrangement (Yes, *n* = 4 vs No, *n* = 12; *p* = 0.92), *MYC* amp with concurrent *BCL2/BC6* rearrangement (Yes, *n* = 5 vs No, *n* = 9; *p* = 0.48). Effect of Hans COO on survival could not be assessed as there was only one evaluable non-GCB case.

## Discussion

Our study shows that *MYC* amp by FISH in aggressive BCLs is rare, occurring only in 0.45% of BCLs in which *MYC* FISH is performed. Two amplification patterns were noted which are cytogentically recognizable, and distinct from lower level copy number gains reported in prior studies. The *MYC* amplification patterns as observed by the *MYC* FISH probes (Fig. 2) are thought to involve an initial excision and formation of unstable extrachromosomal elements, termed double minutes (DM), which are acentric fragments that are amplified individually (29). The DM can reintegrate in a linear fashion into various chromosomal locations and further amplify as a more stable homogenous staining region (HSR) (30). DMs may transform spontaneously into an HSR or after addition of chemotherapeutic drugs (31). The predominance of double minutes (CLD) seen in our cohort of *MYC* amp cases may reflect either relatively recent-onset of amplification or the fact that patients had not yet received treatment.

In describing this cytogenetically distinct FISH pattern, we found *MYC* amp to occur independently (41%), but also often together (59%) with *MYC* rearrangement. In some cases, *MYC*-IGH dual-fusion probe confirmed a *MYC*-IGH rearrangement, but in others *MYC* rearrangement with an unknown partner gene was presumed due to only a portion of the *MYC* probe showing amplification. Using the most widely accepted *MYC* BAP (Abbott Molecular), *MYC* amp may be seen in either the 5′ only probe (55%), the intact probe (40%) or the 3′ only probe (5%). Furthermore, although in most cases *MYC* amp is noted in both the BAP and D-FISH probes, as Table 2 illustrates, some cases showed amp in only the BAP or only D-FISH probe. This heterogeneity likely underscores the complexity of the genomic mechanisms underlying *MYC* aberrations in B cell lymphoma. In addition, it reinforces the need for pathologists, cytogeneticists, and hematologists to understand the differences between different FISH probe sets (Fig. 1). While several promoters have been identified upstream of the *MYC* gene, enhancers have also been identified in the 3′ region^21,22^.

In addition to *MYC* amp, a significant proportion of cases also exhibited *BCL2* (37%) or *BCL6* (7%) rearrangements. Overall, *BCL2* rearrangements were much more common than *BCL6* rearrangements, likely reflecting that the majority of our cases were GCB phenotype^23^. Although more than half of the cases (59%) had a co-existing *MYC* separation in addition to amp, only 29% of the cases met the WHO criteria for DHL/THL.

BCL cases with *MYC* amp are pathologically characterized by a predominance of large cell cytology, unlike DHL/THL which has a predominance of high grade cytologic features^16^. *MYC* amp DLBCLs were also more often GCB phenotype (88%) similar to that seen in BCLs with *MYC* translocation (64% GCB) and DHL/THL (99% GCB)^23^. One prior study observed increased prognostic significance of *MYC* copy number gains in GCB-like cells in comparison to non-GCB^5^. This study, in conjunction with our findings, suggests that *MYC* amp may represent an alternative means of *MYC* dysregulation in the GCB oncogenic pathway.

As has been shown in *MYC*-rearranged BCLs, cases with *MYC* amp usually, but not always, show high *MYC* expression (>40%) by IHC^16^. In this study, 68% of cases had ≥40% *MYC* expression. The lack of universal positivity raises questions about the functional significance of these genetic alterations^10^. Although technical considerations including variable IHC methods, antibodies, interpretation, and cut-off values likely account for some heterogeneity, there is undoubtedly a poorly understood biologic factor as well. At least one study has shown that cases of DHL/THL with absent *MYC* and *BCL2* expression may demonstrate an improved survival in comparison to DHL/THL with double-expression^24–26^. Whether the MYC non-expressing cases may therefore represent nonfunctional *MYC* gene amp deserves further study.

Although the clinical presentation in our series was heterogeneous, it is interesting to note the predominant extranodal involvement (90%), specifically gastrointestinal (32%), identified in our cohort with *MYC* amp. There have been prior reports suggesting associations of other gene amplifications (such as *REL*) with extranodal presentation^7^. Further studies are needed to understand differences in biology of DLBCL presenting as nodal versus extranodal disease as multiple extranodal site involvement is an established clinical prognostic factor in DLBCL^27^.

In our study, *MYC* amp did not appear to have a negative prognostic impact on survival in DLBCL as we found the OS of DLBCL patients with *MYC* amp from our cohort to be no different from the OS of DLBCL patients without *MYC* amp from the larger MER cohort. Similarly, the OS of DHL patients with additional *MYC* amp (from our cohort) was no different from DHL cases without *MYC* amp from the MER suggesting that the presence of *MYC* amp does not worsen the prognosis of *MYC* and *BCL2/BCL6* rearranged DHL. There has been significant debate surrounding the definition and clinical implication of the finding of increased *MYC* copy number by FISH. In 2008, Yoon et al. reported that increased *MYC* copy number (defined as >3 signals, *N* = 11 cases) and/or *MYC* translocation specifically in GCB subtype of DLBCL was associated with a shorter survival (median OS 42 months)^5^. Similar outcomes were reported by Stasik et al. who found a significant correlation between increased *MYC* copy number (defined as >2.2 signals/nucleus) and MYC mRNA expression, higher proliferation and poor outcome (2-year OS 48%)^4^. However, a study by Valera et al. suggested that *MYC* “amplifications” (>4 copies) but not gains (3–4 copies) were associated with unfavorable survival outcomes. The 2-year OS of 80% in our series is in line with other studies that did not find low-level *MYC* copy number increases to be prognostic^2^.

Our study distinguishes itself from the published literature by focusing on cases with uncountable *MYC* signals on FISH and excluding low-level copy number gains. In relation to prior publications, the number of cases with *MYC* amplified DLBCL in our study is large. The hypothesis that *MYC* amp is a negative prognostic factor was not supported by our findings. In fact, our results support the current WHO criteria for DHL/THL that includes only *MYC* rearrangements and excludes *MYC* amp from its definition. These data have important implications in clinical practice where FISH is routinely used to identify the DLBCL cases requiring intensive chemotherapy. Based on our results, we believe that the finding of *MYC* amp alone on FISH should not be interpreted as a marker of aggressive disease and patients should continue to be treated with the standard of care first-line regimen as for DLBCL i.e., currently R-CHOP^28^. Unfortunately, it is hard to draw a conclusion regarding the prognostic significance of finding *MYC* amp (without rearrangement) in conjunction with *BCL2* and/or *BCL6* rearrangements since the numbers in our cohort were small.

Our study is not without limitations such as its retrospective nature, sample size, heterogeneity of treatment, selection bias and missing data. Nevertheless, to the best of our knowledge, this is the largest known cohort of BCLs with *MYC* amp from a large reference laboratory. Numerous interesting observations have come to light from our characterization of this rare but distinct group of cases with *MYC* amp on FISH in aggressive BCLs. The most important being that *MYC* amp alone does not appear to impact outcomes in DLBCL patients treated with standard anthracycline based regimens. Hopefully, larger, multi-center, patient cohorts with a similar definition of *MYC* amp will be studied to validate these data, perhaps focusing on cohorts of extranodal large cell lymphoma to enrich for these cases. In addition, future research should investigate the incidence and significance of other amplification in other commonly used FISH probes such as *BCL2* and *BCL6* in BCL. Finally, given the frequency of lower-level copy number alterations in DLBCL, and conflicting reports of their significance, additional studies are needed in this area as well.

## Supplementary information

Supplemental figure legend

Supplemental Figure
