# Supplementary material for: High level MYC amplification in B-cell lymphomas: is it a marker of aggressive disease?
Source: Blood Cancer J. 2020 Jan 13;10(1):5. doi: 10.1038/s41408-019-0271-z (PMC6957498; doi:10.1038/s41408-019-0271-z)
Supplement: Supplementary file 1 — Supplemental figure legend [file 41408_2019_271_MOESM1_ESM.docx]

**Supplemental Figure:** Overall survival of excluded patients with MYC amp [*MYC* amp at lymphoma relapse (N=5), non-DLBCL morphology (N=5; 3 HGBCL; 2 plasmablastic lymphomas) and transformation from CLL or FL with prior anthracycline use (N=3)] compared to included patients.
