# Supplementary figures and images for: High level MYC amplification in B-cell lymphomas: is it a marker of aggressive disease?
Source: Blood Cancer J. 2020 Jan 13;10(1):5. doi: 10.1038/s41408-019-0271-z (PMC6957498; doi:10.1038/s41408-019-0271-z)

# Overall Survival Patients Included vs. Excluded in Analysis

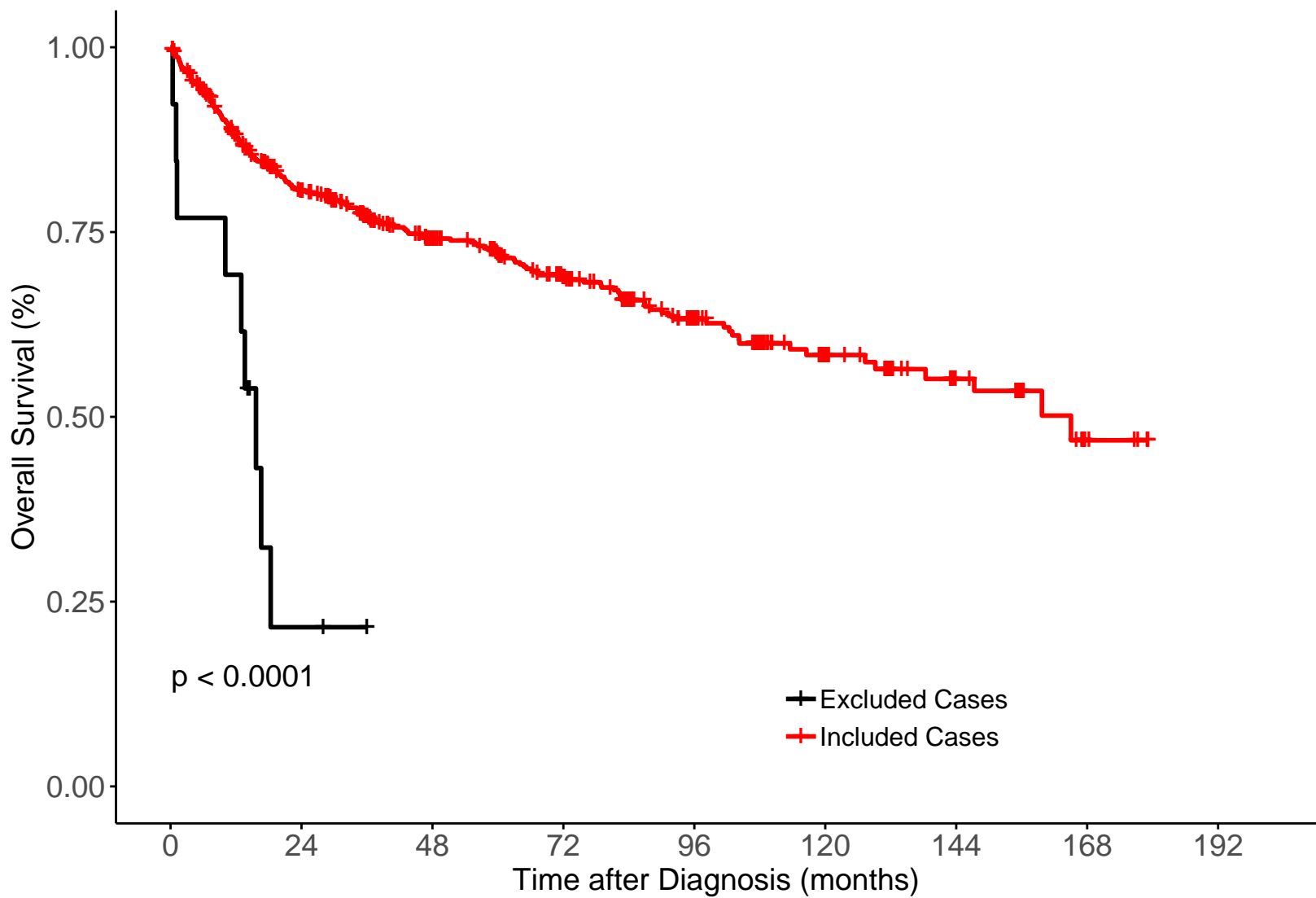

Supplement: Supplementary file 2 — Supplemental Figure [file 41408_2019_271_MOESM2_ESM.pdf]
